# Career calling, ethical sensitivity, and decision-making ability in intensive care nurses: a mediating effect model

**DOI:** 10.3389/fpsyg.2025.1512533

**Published:** 2025-03-24

**Authors:** Ke Wang, Yuanyuan Mi, Yanli Wu, Huimin Sun

**Affiliations:** ^1^Zhongnan Hospital, Wuhan University, Wuhan, China; ^2^Union Hospital, Huanzhong University of Science and Technology, Wuhan, Hebei, China

**Keywords:** intensive care, ethical sensitivity, career calling, nurses, ethical decision-making

## Abstract

**Aims:**

To investigate the simultaneous effects of ethical sensitivity and career calling on intensive care nurses’ decision-making ability.

**Methods:**

A total 361 intensive care nurses in Hubei Province were selected as survey subjects. The survey employed a general information questionnaire, a nurse career calling scale, a Chinese version of the nursing ethics decision-making ability questionnaire, and an ethics sensitivity questionnaire. A structural equation model was constructed using the AMOS software.

**Results:**

The ethical decision-making ability of intensive care nurses earned a score of (267.62 ± 28.15). Ethical sensitivity (*r* = 0.584, *p* < 0.001) and career calling (*r* = 0.566, *p* < 0.001) positively correlated with ethical decision-making ability in nursing. Career calling partially mediates between ethical sensitivity and nursing ethical decision-making ability, with a mediation effect of 0.246.

**Conclusion:**

Intensive care nurses exhibited moderate ethical decision-making ability and require further improvement. Career calling partly mediates between moral sensitivity and nursing ethical decision-making ability. Enhancing ethical sensitivity and career calling can help improve ethical decision-making ability among intensive care nurses.

## 1 Introduction

Intensive care nurses routinely manage patients with complex conditions, encountering high levels of nursing difficulty and experiencing numerous ethical dilemmas in clinical practice. Research indicates that up to 80% of intensive care nurses have encountered ethical dilemmas at least once in their careers (Hickey, 2022). An ethical dilemma, also referred to as a moral dilemma, arises when intensive care nurses face constraints due to shortages of medical equipment and human resources, lack the authority to voice their concerns, and are unable to act following their moral principles (Xu et al., 2024). Consequently, they may provide care that prolongs physiological pain and undermines the dignity of critically ill or dying patients (Cooke et al., 2022). This situation leads to negative mental and physical health outcomes for intensive care nurses, such as depression, anger, sadness, guilt, anxiety, fatigue, and palpitations (Jameton, 2017). Ethical decision-making for nurses refers to the process by which nurses determine the optimal nursing plan based on professional theory and experience, considering the actual clinical situation. This process integrates investigation and scientific thinking from a series of alternative plans (Yang et al., 2022). Effective ethical decision-making is essential to providing high-quality care and ensuring ethical nursing practice (Alzghoul and Jones-Bonofiglio, 2020). Compared with nurses in general wards, intensive care nurses encounter ethical decision-making more frequently (Xu and Zhang, 2022). The closed working environment and the specialized nature of patient care in the Intensive Care Unit (ICU) also contribute to increased negative emotions among intensive care nurses. These factors can adversely affect their ability to make ethical decisions in clinical practice (Jia et al., 2017). Nurses lacking ethical decision-making skills are prone to errors, affecting nurse–patient relationships, nursing quality, and management efficiency. Previous researches indicated a negative correlation between ethical decision-making ability and occupational burnout among intensive care nurses (Wang et al., 2021).

Jia et al. (2017) demonstrated that the ethical decision-making abilities of intensive care nurses are at a moderate level, falling short of high-level standards. Occupational fatigue, high work pressure, rigid nursing requirements, a large volume of critically ill patients, and other factors can adversely affect the capacity of nurses to make ethical decisions. The higher the negative emotional level of clinical nurses, the more vulnerable their ethical decision-making ability is negatively affected (Jia et al., 2017). When the mental health level of clinical nurses declines, their ethical decision-making ability will be reduced (Yang et al., 2022). Ethical sensitivity refers to the awareness of ethical issues despite the absence of obvious ethical conflicts. It includes responding to the needs of others, anticipating the potential effects of certain actions on others, and determining whether actions violate internal ethical or behavioral guidelines. Ethical sensitivity is a prerequisite for participating in nursing ethical decision-making and executing ethical actions (Yue et al., 2021). If individuals fail to identify ethical issues effectively, they cannot make ethical judgments or take positive actions (Yue et al., 2021). Ethical sensitivity has been shown to predict nursing ethical decision-making ability positively; some scholars revealed that improving the ethical sensitivity of nurses can enhance the sense of professional benefits of nurses (Chen et al., 2020). However, its underlying mechanism remains unclear career calling represents the passion and motivation of individuals for their work. The National Health Commission has highlighted the need to strengthen the construction of a critical care professional team. It regards critical care nurses as urgently needed and promotes the expansion of critical care nurses. However, intensive care nurses are among the professionals in the healthcare industry with a high turnover rate and a high incidence of occupational burnout. Previous studies have shown that career calling, as a positive work value, significantly reduces nurse burnout (Zhu et al., 2023). Career calling can motivate nurses to remain engaged in their work despite demanding environments (Zhu et al., 2023). Mao Yunhua et al. (2019) and Shen and Qian (2022) also demonstrated that stimulating a sense of career calling in nurses can improve job satisfaction and reduce the turnover of skilled nursing professionals. Intensive care nurses, a high-risk group for occupational burnout and negative emotions (Qu et al., 2020), have not been studied for their degree of career calling. Career calling can influence individuals’ professional behavior and practical decision-making (Cui and Su, 2024), which has the attribute of positive emotion.

Given this, the current study constructs a Structural Equation Model (SEM) using career calling as a mediating factor to explore the relationship between ethical sensitivity and nursing ethical decision-making ability, providing ideas for improving ethical decision-making ability from the perspective of positive psychology among intensive care nurses.

## 2 Methods

### 2.1 Research design

A cross-sectional and questionnaire-based design was used. The study was conducted following the principles of the Declaration of Helsinki, developed by the World Medical Association (2013). Nurses’ participation was voluntary and informed consent was obtained before administration of the questionnaire. All data were collected anonymously.

### 2.2 Participants

This study employed convenience sampling to select intensive care nurses as research subjects from 8 Class A tertiary hospitals in Wuhan between August and December 2023. The inclusion criteria were ① at least 1 year of ICU work experience ② informed consent and voluntary participation in the study. The exclusion criteria required ① nurses on external study programs or hospital-based refresher courses and ② those on sick or personal leave. A convenience sample of 366 intensive care nurses took part in the study.

According to Kline (2016), although there is no simple rule of thumb about the suitable sample size for SEM, 20 cases may be considered suitable per parameter. Following Kline’s rule, the required sample size in this study would be 100 cases. Here, the sample size was equal to 366 nurses exceeding the minimum sample size.

### 2.3 Instruments used in the study

#### 2.3.1 General information survey

Drawing on an extensive literature review and in accordance with the specific aims of this study, the researcher developed a demographic survey form. The form primarily collected data on gender, age, working years, marital status, educational level, type of hospital, personnel status, and night shift per month.

#### 2.3.2 Revised Chinese moral sensitivity questionnaire

This scale was developed by Lützén et al. (2006) and then translated into Chinese by Huang et al. (2015) after rigorous cultural adaptation. It comprises two dimensions, ① moral responsibility and strength and ② moral burden, with a total of nine items. The scale uses a 6-point Likert scale (1 = strongly disagree, 6 = strongly agree), with total scores ranging from 9 to 54; higher scores indicate greater ethical sensitivity among nurses. In this study, the Cronbach’s alpha coefficient for the scale was 0.78.

#### 2.3.3 Career calling questionnaire

This scale was developed by Dobrow and Tosti-Kharas (2011) and then translated into Chinese by Pei and Zhao (2015). Unidimensional and consisting of 12 items, it uses a 7-point Likert scale (1 = strongly disagree, 7 = strongly agree). Higher scores indicate a stronger subjective identification with professional values and a greater experience of intrinsic passion for the job. This scale has been extensively applied in various research contexts, both domestically and internationally, to assess career calling among ICU nurses. In this study, the Cronbach’s alpha coefficient for the scale was 0.96.

#### 2.3.4 Judgment about nursing decisions (JAND)

Developed by Ketefian (1981) and revised in 2007, this scale was translated into Chinese by Zhang Huiying et al. (2016). The Chinese version demonstrated a reliability index of 0.745 and a validity index of 0.982. The questionnaire comprises 6 ethical dilemma stories, each followed by 6–7 questions, resulting in a total of 37 questions. Participants respond to each question in two stages: the first stage assesses ethical decision-making, reflecting the actions nurses would ideally take without external constraints; the second stage involves ethical implementation, representing the behaviors nurses would adopt in actual scenarios, considering practical constraints. The combined score for both stages ranges from 55 to 185, with higher scores indicating enhanced capabilities for ethical decision-making. In this scoring system, “recommended” items are assigned scores of 5, 4, 3, 2, and 1; “not recommended” items are assigned scores of 1, 2, 3, 4, and 5; and “unclear” items are assigned scores of 3, 4, 5, 4, and 3. The total score for the questionnaire, combining both the first and second stages, ranges from 110 to 370, with higher scores reflecting a higher level of ethical decision-making ability among nurses. Scores below 222 indicate a low level of ethical decision-making ability, scores between 222 and 296 are considered moderate, and scores above 296 indicate a high level of ethical decision-making ability. In this study, the scale exhibited a Cronbach’s alpha coefficient of 0.867.

### 2.4 Data collection

The survey via Wenjuanxing required that all mandatory fields be filled in for successful submission. A survey team was established, with each selected hospital represented by a member from its nursing department. Team members distributed QR codes for the survey, ensuring that all questions were mandatory. Each IP address was limited to one submission. The purpose, completion instructions, and guidelines of the survey were explained to the nurses by using consistent and clear language, ensuring that they understood the survey is anonymous and encouraging them to provide truthful responses.

### 2.5 Data analysis

Data were analyzed using SPSS 22.0 (IBM, Armonk, NY, USA) and Amos 25.0. The quantitative data in this study were normally distributed, as verified by normality testing, and described using the mean ± standard deviation. Qualitative data were described using frequencies and proportions. The Harman single-factor test was used to assess common method bias. The relationships between variables were analyzed using Pearson correlation. A structural equation model was constructed with AMOS 25.0 to validate the hypotheses developed in this study. The mediating effect of career calling was tested using the Bootstrap method, where an estimated effect is considered valid if its 95% confidence interval excludes zero.

## 3 Results

### 3.1 General information on ICU nurses

A total of 366 electronic questionnaires were collected in this study. After excluding 5 invalid questionnaires with the same answer number for each option, 361 valid questionnaires remained, resulting in an effective response rate of 98.6%. Of the 361 nurses, 268 (74.23%) were female, accounting for the vast majority of the nurses group. 219 (60.66%) ICU nurses were under 25 years old, 223 (61.77%) ICU nurses worked for <6 years, meaning the ICU nursing team tended to be younger. The marital status of 281 (77.84%) nurses was unmarried. 327 (90.58%) ICU nurses had a bachelor’s degree or above.285 (78.95%) ICU nurses were from general hospitals.255 (70.64%) of the ICU nurses were contract workers.186 (51.52%) ICU nurses had least 7 night shifts every month. Specific information can be found in Table 1.

**Table 1:** General Information on ICU nurses (*N* = 361).

Variables	Categories	*n* (%)
Gender	Male	93 (25.76)
	Female	268 (74.23)
Age (years)	<25	219 (60.66)
	≥25	142 (39.34)
Years of working	1–<6	223 (61.77)
	6–10	88 (24.38)
	>11	50 (13.85)
Marital status	Unmarried	281 (77.84)
	Married	80 (22.16)
Education level	Associate degree	34 (9.42)
	Bachelor degree	309 (85.60)
	Master’s degree	18 (4.98)
Type of hospital	General hospital	285 (78.95)
	Specialized hospital	76 (21.05)
Personnel status	Permanent staff	67 (18.56)
	Contract staff	255 (70.64)
	Others	39 (10.80)
Night shift per month	0	28 (7.76)
	1–3	29 (8.03)
	4–6	118 (32.69)
	≥7	186 (51.52)

### 3.2 Common method deviation

The Harman single-factor test was used to assess for the presence of common method bias. The results showed that a total of 6 common factors with eigenvalues >1 were extracted, and the first common factor comprised 36.33% of the variance—that is, less than the critical value of 40%. Therefore, common method bias is not a significant factor in this study.

### 3.3 Scores for ethical sensitivity, career calling, and ethical decision-making ability among ICU nurses

The results of this study showed that the intensive care nurses exhibited an ethical sensitivity score of 41.39 ± 9.49, an ethical decision-making ability score of 267.62 ± 28.15, and a career calling score of 48.85 ± 8.93 (Table 2).

**Table 2:** Total scores and dimension scores for ethical sensitivity, career calling, and ethical decision-making ability among ICU nurses (*n* = 361), *^−^x ± s.*

Questionnaire	Item	Number of entries	Scores Mean ± SD	Equal distribution of items Mean ± SD
Ethical sensitivity	Moral responsibility and power	5	25.55 ± 0.45	5.11 ± 0.54
	Moral burden	4	15.84 ± 0.52	3.96 ± 0.58
Career calling	Total score	12	48.85 ± 8.93	4.07 ± 0.46
Ethical decision-making ability	Ethical choice	37	146.68 ± 16.23	3.97 ± 0.67
	Ethical Action	37	120.37 ± 10.52	3.25 ± 0.32

### 3.4 Correlation analysis of ethical sensitivity, ethical decision-making ability, and career calling among ICU nurses

The total scores for ethical sensitivity, career calling, and ethical decision-making ability positively correlated with the dimension scores (Table 3).

**Table 3:** Correlation (*r*-value) between career calling, ethical sensitivity, and ethical decision-making ability among ICU nurses.

Item	①	②	③	④	⑤	⑥	⑦
①	1	–	–	–	–	–	–
②	0.253	1	–	–	–	–	–
③	0.564	0.322	1	–	–	–	–
④	0.449	0.335	0.566	1	–	–	–
⑤	0.566	0.584	0.424	0.522	1	–	–
⑥	0.482	0.352	0.339	0.204	0.343	1	–
⑦	0.463	0.375	0.429	0.584	0.425	0.684	1

① Total score for career calling; ② Total score for ethical sensitivity; ③ Moral Responsibility and Power; ④ Moral burden; ⑤ Total score for ethical decision-making ability; ⑥ Ethical choice; ⑦ Ethical behavior; *P <* 0.05.

### 3.5 The mediating role of career calling in ethical sensitivity and ethical decision-making ability among ICU nurses

**Figure 1:**
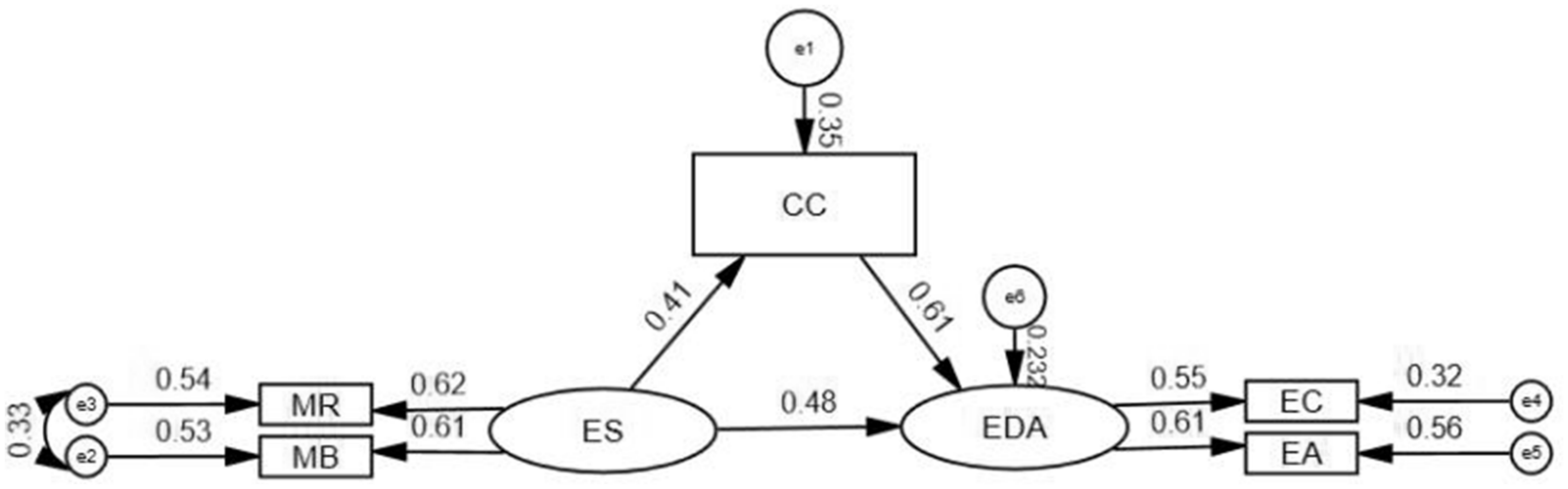
Mediating effects model of career calling, ethical sensitivity, and ethical decision-making ability among ICU nurses (standardized). CC, career calling; ES, ethical sensitivity; EDA, ethical decision-making ability; MR, moral responsibility and strength; MB, moral burden; EC, ethical choice; EA, ethical action.

A structural equation model was developed, with ethical sensitivity as the independent variable, nursing ethical decision-making ability as the dependent variable, and career calling as the mediating variable. A mediating effect model of career calling was ultimately formed between ethical sensitivity and ethical decision-making among intensive care nurses. The model demonstrated a good fit, with specific parameters in Table 4 and all indicators meeting the reference standards. The validity of the model is illustrated in Figure 1. To assess the mediating effect, we used Bootstrap sampling with 5,000 repetitions. The confidence intervals for the total effect (95% CI: 0.611–0.833), indirect effect (95% CI: 0.122–0.312), and direct effect (95% CI: 0.352–0.575) of ethical sensitivity on nursing ethical decision-making ability did not include 0. This result indicates that career calling partly mediates the relationship between ethical sensitivity and nursing ethical decision-making ability, with the mediating effect constituting 34.07% (Table 5).

**Table 4:** Standards and results for fitting indices in structural equation modeling.

Fit index	*Χ^2^/df*	*CFI*	*AGFI*	*GFI*	*NFI*	*RMSEA*	*TLI*
Reference value	1.000–2.000	>0.900	>0.900	>0.900	>0.900	<0.080	>0.900
Actual value	1.067	0.96	0.93	0.95	0.96	0.06	0.92

**Table 5:** Decomposition of the effect of career calling on ethical sensitivity and nursing ethical decision-making ability (*n* = 361).

Item	Standardized coefficient	*SE*	95% CI	*p*	Proportion of effect (%)
Indirect effect	0.246	0.042	0.122–0.312	<0.001	34.07
Direct effect	0.476	0.072	0.352–0.575	<0.001	65.93
Total effect	0.722	0.045	0.611–0.833	<0.001	100

## 4 Discussion

The results of this study showed that intensive care nurses had an average ethical sensitivity score of 41.39 ± 9.49, which exceeded the median score of the scale and was consistent with Borhani et al. (2017) and slightly higher than those reported by Yue et al. (2021) and He et al. (2023). Yue et al. (2021) reported on nurses from county-level hospitals, whereas He et al. (2023) surveyed psychiatric nurses. The nature of hospitals can affect nurses’ ethical sensitivity (Yue et al., 2021). The current study surveyed ICU nurses from provincial capital hospitals, who are typically more exposed to nursing ethics training and encounter more complex ethical dilemmas because of the severity of patient conditions. The average scores for dimensions such as moral responsibility and strength were 5.11 ± 0.54, higher than for moral burden (3.96 ± 0.58). The potential factor may be the long-standing traditional Chinese values, which promote strong moral responsibility and have been ingrained in Chinese culture over thousands of years. This background likely contributes to the increased sense of responsibility and ethical sensitivity of the ICU nurses.

The nursing ethical decision-making ability score of intensive care nurses was (267.62 ± 28.15), the ethical choice dimension score was (147.25 ± 16.23), and the ethical action dimension score was (120.37 ± 10.52). These scores indicate a moderate level of ethical decision-making ability—that is, lower than the results obtained by Xu and Zhang (2022) and Wang et al. (2021). This difference may be related to the length of service of the participants. Ethical issues are particularly prominent in ICU settings, where nurses face ethical decision-making situations more frequently than nurses in other departments. Nonetheless, in the current study, 61.77% of intensive care nurses had <5 years of working and most were still in the early stages of their careers. Working years can affect the ethical decision-making ability of intensive care nurses in nursing (Hu et al., 2023). These nurses generally lack experience in managing ethical issues in clinical practice and struggle to handle complex ethical issues calmly. In addition, the training mode for nurses in China focuses on exam-oriented education, hence the strong theoretical knowledge yet inadequate practical skills. In the current study, the score for the ethical choice of intensive care nurses exceeded that for their ethical action, suggesting that despite their high level of knowledge in nursing ethics decision-making, intensive care nurses exhibit a relatively low level of correct ethical behaviors when facing ethical dilemmas in clinical practice. This finding further emphasizes the need for managers and universities to strengthen training in practical ethical skills.

The score of intensive care nurses is (48.85 ± 8.93), which exceeds the median score of the scale but is lower than the score reported by Shen and Qian (2022) on 440 oncology nurses. It may be related to the different care recipients, and the work environment can affect the career calling level of nurses (Ni et al., 2023). This result may be related to the employment duration of the participants. In the current study, 61.77% of intensive care nurses had been employed for 1 to <6 years. Despite their high expectations for their work and future, they are prone to negative emotions due to limited clinical experience, communication skills, and psychological resilience. Consequently, their sense of career calling is not high. In addition, intensive care nurses tend to care for critically ill patients, with long-term emotional investment and frequent exposure to patient death. This environment renders them susceptible to psychological issues, such as emotional exhaustion, decreased sense of career calling, and compassion fatigue, further lowering their sense of career calling. In addition, the frequency of night shifts is also a reason for the low level of career calling among intensive care nurses (Mao Yunhua et al., 2019). In this study, over 50% of intensive care nurses had at least 7 night shifts per month. Night shift nurses often need to care for more patients, and their overloaded work and prolonged sleep deprivation can easily cause emotional tension and physical fatigue (Guo Yifeng et al., 2021). Night shift nurses need to handle sudden events such as changes in the patient’s condition alone, which can easily lead to a sense of powerlessness, causing excessive psychological burden and ultimately resulting in a low level of career calling.

The results of this study demonstrate a positive correlation between ethical sensitivity and ethical decision-making capability among intensive care nurses. This finding aligns with existing literature (He et al., 2023), indicating that higher levels of ethical sensitivity among intensive care nurses are associated with more effective implementation of nursing ethical decisions. Intensive care nurses with high ethical sensitivity are skilled at identifying and addressing ethical issues that arise in patient care, effectively responding in a manner that prioritizes patient needs. In addition, ethical sensitivity is positively correlated with career calling among ICU nurses, indicating that those possessing greater ethical sensitivity are more likely to be engaged in their work, and exhibit higher levels of identification and loyalty to their profession (Yue et al., 2021), and experience a stronger sense of value and meaning in their roles. Consequently, these factors contribute to a higher level of Career calling. Career calling is positively correlated with ethical decision-making in nursing, likely because nurses with high career calling levels have a positive work attitude and a high sense of responsibility. When addressing ethical issues, they can respond proactively and make informed sound judgments. Research shows that a positive work attitude and a strong sense of responsibility enhance decision-making in both clinical and ethical contexts (Kim et al., 2015). The study also revealed that positive nursing professional values can enhance decision-making skills and overall nursing quality (Poorchangizi et al., 2017). Thus, ICU managers should focus on helping nurses improve their career calling level and approach ethical issues with a positive attitude.

The mediating effect structure of this study shows that career calling mediates between ethical sensitivity and nursing ethical decision-making ability in intensive care nurses. Ethical sensitivity directly affects ethical decision-making ability and indirectly influences ethical decision-making ability through career calling. Compared with other nurses, ICU nurses with high ethical sensitivity exhibit a higher ability to identify ethical issues, reflect on their professional values and roles, and deepen their understanding of their roles and responsibilities. These attributes enable them to promote their comprehensive consideration of the needs of patients and their families to make appropriate ethical decisions in nursing. Research has shown that ethical sensitivity is a crucial component of clinical ethical decision-making and high-quality healthcare (Palazoglu and Koç, 2019). Various ethical issues or dilemmas may arise in the workplace, potentially leading to negative outcomes, such as job burnout (Palazoglu and Koç, 2019). However, nurses with a high level of career calling will not perceive these concerns negatively. Owing to their firm professional beliefs, they incorporate their ethical knowledge, consider problems dialectically, and make reasonable ethical decisions in nursing.

Nursing managers can enhance the ethical decision-making ability of intensive care nurses by improving their career calling and ethical sensitivity. Based on the internal and external factors that affect career calling, nursing managers can regularly organize team building activities and group mindfulness stress reduction activities to strengthen team cohesion and nurses’ psychological resilience, reduce the work pressure of intensive care nurses, and enhance their sense of career calling. Previous research found that improving the working environment is beneficial for enhancing nurses’ autonomy and intrinsic motivation (Zhang et al., 2019). Nursing managers can also enhance the intrinsic motivation and confidence of intensive care nurses by implementing flexible scheduling, optimizing human resource allocation, implementing salary incentive systems, and providing career development opportunities for nurses, thereby improving their career calling level. In addition, Choe et al. (2020) found that holding ethics seminars is beneficial for enhancing the ethical sensitivity of clinical nurses. Nursing managers can regularly hold ethics seminars to develop conference topics that include nursing ethics, ethical thinking, and ethical issues encountered by ICU nurses in clinical work. This will enhance the ethical thinking and sensitivity of intensive care nurses, and ultimately improve their ability to make ethical decisions in clinical practice. Based on demographic and sociological factors that affect the ethical sensitivity of ICU nurses, such as gender, age, and working years, nursing managers can also provide personalized nursing interventions based on the characteristics of the nursing population. In addition, ethical dilemmas in clinical practice can be reproduced through scenario simulations and case studies, enhancing intensive care nurses’ understanding and practice of ethical decision-making processes, gradually cultivating nurses’ ethical sensitivity, strengthening nurses’ mastery of basic ethical theories, ethical decision-making related knowledge, and application skills, thereby improving their nursing ethical decision-making abilities.

## 5 Limitations

A limitation of this study is that the sample predominantly comes from Hubei Province, restricting its representativeness. In future research, the sample size should be increased, and more diverse regions should be included to enhance the generalizability of the research results. In addition, longitudinal and intervention studies should be conducted to further explore the relationships between ethical sensitivity, career calling, and nursing ethical decision-making ability. This approach is expected to enhance the ethical decision-making ability of intensive care nurses and their capacity to effectively address ethical issues in clinical practice.

## 6 Conclusion

The results of this study suggest that intensive care nurses demonstrate moderate to high levels of ethical sensitivity and moderate levels of career calling and ethical decision-making abilities. Career calling partially mediates between ethical sensitivity and decision-making ability in intensive care nurses. Nursing managers can promote ethical decision-making skills in intensive care nurses by improving ethical sensitivity and reinforcing career calling, encouraging alignment of personal values with the significance of their work, both individually and within their departments.

## Data Availability

The datasets presented in this study can be found in online repositories. The names of the repository/repositories and accession number(s) can be found in the article/supplementary material.
